# Prevalence of osteoporosis and its association with serum vitamin D level in older people in Amirkola, North of Iran 

**Published:** 2012

**Authors:** Zahra Pourhashem, Mohammadali Bayani, Hajighorban Noreddini, Ali Bijani, Seyed Reza Hosseini

**Affiliations:** 1 Babol University of Medical Sciences, Babol, Iran.; 2 Department of Internal Medicine, Ayatollah Rouhani Hospital Babol University of Medical Sciences, Babol, Iran.; 3 Department of Radiology, Babol University of Medical Sciences, Babol, Iran.; 4 Social Health Research Center, Babol University of Medical Sciences, Babol, Iran.

**Keywords:** Osteoporosis, Vitamin D, Bone density, Elderly

## Abstract

**Background:**

Ageing of population worldwide will be responsible for a major increase of the incidence of osteoporosis in elderly. For the individual, osteoporotic fractures result in great disability as well as loss of quality of life and also represent an enormous burden for healthcare systems. This study was conducted to determine the prevalence of osteoporosis and its association with serum vitamin D level in elderly people in Amirkola, North of Iran.

**Methods:**

In this cross-sectional study, 193 subjects aged 60 years old and over were assessed in Amirkola. Using a standard questionnaire, individual characteristics and densitometry (with DXA) results were collected and the data were analyzed with SPSS, chi square tests and linear regression.

**Results:**

The overall prevalence rate of osteoporosis was 32.1% in at least one measurement sites (28.5% in lumbar and 14.5% in femoral region), while 49.7% of elderly people suffering from decreased bone mass (osteopenia). Osteoporosis prevalence in women was 55.7% and this value in elderly men was 12.4%. Bone mineral density has negative association with age in femoral region (p<0.01, r-0.3), but no statistical relationships were seen between bone mineral density and serum 25-hydroxy vitamin D level in this study.

**Conclusion:**

Our findings revealed higher prevalence of osteoporosis in elderly people especially in women compared to other studies in Iran, and also showed high prevalence rate of vitamin D deficiency. No relationships were found between serum vitamin D and bone density in this study.

Osteoporosis is a global problem which is increasing in significance as the population of the world both is growing and ageing. For the individual fragility, fractures result in great suffering, disability as well as loss of productivity and quality of life, fractures also represent an enormous burden for healthcare systems. Older people who suffer hip fractures are often faced with long-term disability that results in loss of independence and higher risk of death. A new audit report by the international osteoporosis foundation (IOF) show that osteoporosis is a serious and growing problem throughout the Middle East (1); but ignored as it competes with other chronic diseases. Notwithstanding the burden of fragility fractures, osteoporosis remains greatly underdiagnosed and undertreated, both health professionals and public awareness is suboptimal in our region. Osteoporosis prevalence is variable throughout the world; and the different factors are responsible for the peak bone mass, such as genetics, race and nutrition. Considering the ageing population of Iran, the different lifestyles of our people and the limited information about this problem in our region, this study was conducted to examine the prevalence of osteoporosis and the relationship between bone mineral density and serum vitamin D level among the elderly people in Amirkola, Babol.

## Methods

This is a part of a large, health survey cohort study among the elderly people of Amirkola [Amirkola Health and Ageing Project (AHAP)]. In this cross-sectional, descriptive-analytical study, people aged 60 years old and over were recruited by calling them personally, randomly selected from the list of elderly people living in this city registered at Amirkola Health Care Center. Using a standard questionnaire, the individual characteristic and demographic data were collected. 

**Bone mineral density**: Bone density was measured by dual energy x-ray absorptiometry with the Lexxos densitometer. BMD results were expressed in absolute values (g/cm2) and T-score for lumbar spine and proximal femur. According to the WHO criteria, osteoporosis is defined as a BMD that lies 2.5 standard deviations or more below the average value for young healthy adults (a T-score of <-2.5 SD) and T-score between -1.0 and -2.5 considered osteopenia.

**Vitamin D Assessment**: Fasting blood sample was obtained for biochemical and hormonal analysis at the time of clinical assessment and vitamin D measurement was done at the laboratory of Cellular and Molecular Research Center of Babol University of Medical Sciences. Vitamin D was assessed using Elisa and vitamin D deficiency is defined as a 25-hydroxy vitamin level of less than 20 ng per milliliter (50 nmol / lit). A level of less than 25-hydroxy vitamin D of 21 to 29 nanogram per milliliter was considered Insufficient and a level of 30 ng per milliliter or greater was considered sufficient.

**Statistical analysis**: Summary statistics, including mean, standard deviation (SD), standard errors (SE), median, and range were calculated for bone mineral density, serum 25(OH)D, PTH and other biochemical studies. The data were analyzed with chi square test and linear regression and p<0.05 was considered significant. 

## Results

One hundred ninety three subjects (54.4% men, 45.6% women) between 60 to 88 years (mean±SD, 68.39±6.71 years) participated in the study. The basic characteristics of the subjects is shown in table1. Table 2 shows the prevalence of osteopenia and osteoporosis in men and women at different sites. 

The mean spinal BMD was 0.93±0.15 in men and 0.77±0.14 in elderly women and mean Femoral BMD among men and women was 0.90±0.15 and 0.79±0.13 respectively (p<0.05). In the patients with osteoporosis 18 subjects (29%) had previous fractures, and 37 (28.2%) with normal bone density had previous fracture as well which does not show statistical relation between osteoporosis and previous fracture. Table 3 shows the correlations between the variables in this study.

Serum parathyroid hormone, 25-hydroxyvitamin D, calcium and phosphorus concentration were not correlated with age and bone mineral density, but serum vitamin D level and parathyroid hormone concentration had negative association (p<0.01, r= 0.26). Femoral bone density was negatively correlated with age but lumbar bone density was not. Table 4 shows the prevalence of osteoporosis with respect to age at least one of the measured sites. Vitamin D status is shown in table 5.

**Table 1:** Base line characteristics of 193 subjects

**p-value**	**Total**	**Male** **(n=105)** **Mean±SD**	**Female** **(n=88)** **Mean±SD**	**Variable**
0.188	68.39±6.17	68.97±7.08	67.69±6.20	Age (year)
0.625	20.35±16.96	19.74±15.02	21.03±18.98	25(OH) D (ng/ml)
0.095	38.01±23.29	35.18±20.39	41.19±25.93	PTH (pmol/l)
0.061	9.33±0.43	9.38±0.44	9.26±0.41	Ca (mmol/l)
0.171	4.04±0.55	3.98±0.56	4.09±0.53	Phosphorus (nmol/l)
0.000	0.85±0.15	0.90±0.15	0.79±0.13	Femur BMD (g/cm^2^)
0.000	0.86±0.15	0.93±0.15	0.77±0.14	Spine BMD (g/cm^2^)
0.000	-1.37±1.18	-1.03±1.19	-1.78±1.03	T-score (Femoral)
0.000	-1.68±1.35	-1.05±1.09	-2.43±25	T-score (Lumbar)

BMD: Bone Mineral Density

Parathormone hormone

**Table 2.** Prevalence of osteopenia and osteoporosis at different sites

**Osteopenia** ٭				**Osteoporosis** ▪		
**Sex**	**Spine** **N (%)**	**Femur** **N (%)**	**One of two** **N (%) **	**Spine** **N (%)**	**Femur** **N (%)**	**One of two** **N (%)**
Female	33 (37.5)	46 (52.3)	31 (35.2)	43 (48.9)	22 (25)	49 (55.7)
Male	43 (41)	50 (47.6)	65 (61.9)	12 (11.4)	6 (5.7)	13 (12.4)
Total	76 (39.4)	96 (49.7)	96 (49.7)	55 (28.5)	28 (14.5)	62 (32.1)

* -2.5< T-score <-1.0,

▪ T-score <-2.5

**Table 3:** Correlations between age, bone mineral density and serum biochemistry

**variable**	**Age**	**Spine BMD**	**Femoral BMD**	**Ca**	**Phosphorus**	**PTH**	**Vitamin D**
Age	-	-0.002	-0.301*	0.02	0.06	0.10	0.04
Spinal BMD	-0.002	-	0.579*	0.68	0.52	-0.11	0.01
Femoral BMD	-0.301*	0.579*	^-^	0.034	0.068	-0.13	-0.031
Ca	-0.20	0.068	0.034	-	0.238*	-0.09	0.04
Phosphorus	0.060	0.052	-0.068	0.238*	-	0.07	-0.03
PTH	0.101	-0.11	-0.13	0.09	0.07	-	-0.266*
Vitamin D	0.046	0.017	-0.031	0.04	-0.03	-0.266*	-

* P < 0.01 (correlation in significant at the 0.01 level (2- tailed))

BMD: Bone Mineral Density

Parathormone hormone

**Table 4:** Prevalence of osteoporosis and osteopenia with respect to age according to WHO reference (spine or femur)

**Age**	**Osteopenia** **N (%)**	**Osteoporosis** **N (%)**	**Total** **N (%)**
60-64	45 (61.6)	17 (23.3)	73 (37.8)
65–74	35 (42.2)	30 (36.1)	83 (43)
75	16 (43.2)	15 (40.5)	37 (19.2)
Total	96 (49.7)	62 (32.1)	193 (100)

**Table 5:** Vitamin D status in 168 subjects

**Vitamin D sufficient** **(≥30ng/ml)** **N (%)**	**Vitamin D insufficient** **(21-29ng/ml)** **N (%)**	**Vitamin D deficient** **(≤20ng/ml)** **N (%)**	**Sex** **N (%)**
9 (11.4)	12 (15.2)	58 (73.9)	Female
7 (7.9)	11 (12.9)	71 (79.8)	Male
16 (9.5)	23 (13.7)	129 (76.8)	Total

**Figure 1:**
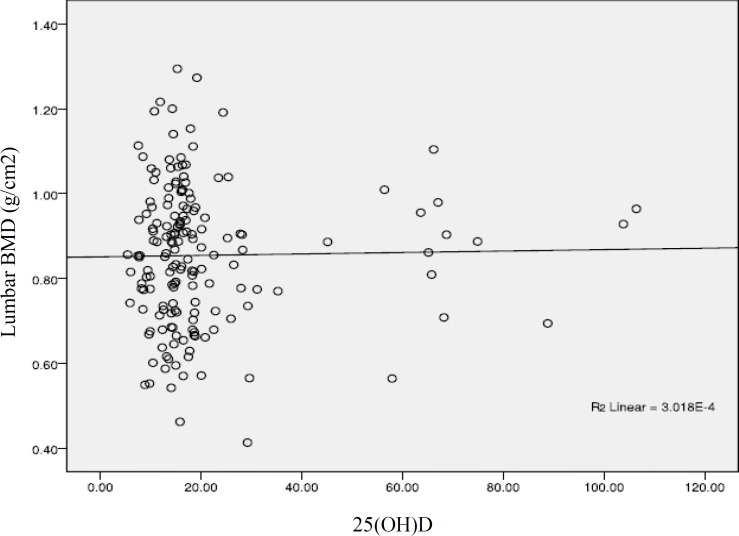
Correlation between serum vitamin D level and lumbar bone mineral density

**Figure 2:**
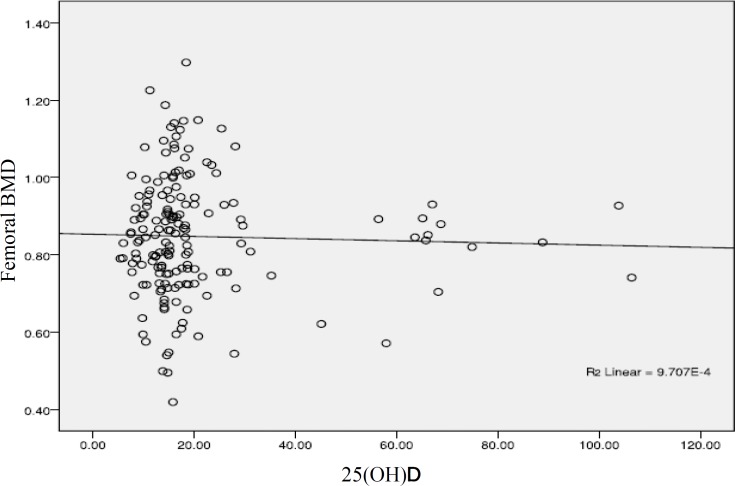
Correlation between serum vitamin D level and femoral bone mineral density

**Table 6:** Osteoporosis prevalence in different studies in Iran

**Study**	**Sample size** **(N)**	**Age**	**Anatomic site**	**Osteoporosis**	**Osteopenia**
Tehran	754	40-60	Spine Femur	15.8% 2.9%	33.8% 26.8%
Kurdestan	305	39-79	Spine Femur	17% 30.8%	56.1% 47.9%
Bushehr	558	20-69	Spine Femur	3.2% 1.5%	23.9% 16.5%

**Table 7:** Osteoporosis prevalence in different countries in Middle East

	**Male**	**Female**
*Iran	17%	28.8%
Turkey	7.5%	33.3%
Saudi Arabia	33.2%	44.5%
*Lebanon	17%	31%

* Osteoporosis prevalence at Lumbar spine

According to Iranian multicenter osteoporosis study

## Discussion

In this study, the prevalence of osteoporosis was 28.5% at lumbar vertebra, 14.5% at femoral region and 32.1% at least in one of the two measured sites. Table 6 and 7 show the prevalence of osteoporosis in different studies in Iran and other countries in our region (2-8). The reasons of higher prevalence rate of osteoporosis in current study, may be of higher than the mean age. In Jamshidian’s study, the prevalence of osteoporosis among 40–60 years old the Tehrani women was assessed (2) and the same study was done in Bushehr between 20–69 years old women (2, 4). In women, prevalence of osteoporosis was 55.7% in at least one of two measured sites, while this value was 12.4% in men. The prevalence of osteoporosis in women in our study was higher than the other studies shown in tables 6 and 7. This might be due to the sedentary lifestyle, worse nutritional program in elderly and high prevalence of chronic diseases (such as hypertension and diabetes) and disability especially in women which is according to a study done in Amirkola to determine the prevalence of chronic diseases among the elderly people (9) . 

The mean spinal BMD in women at lumbar and femoral regions was 0.77±0.14 and 0.79±0.13, respectively, and these results in men were 0.93±0.15 and 0.90±0.15, which shows significant statistical difference between men and women (p<0.01), similar to the findings of Iranian multicenter osteoporosis study which shows the higher prevalence of osteoporosis in women (5). Like the other studies, our findings show that increasing age is associated with bone density decrement and increasing prevalence of osteoporosis (10, 11). In this study, the negative association between bone density and age is seen only in femoral region; this is in concordance with higher incidence of hip fracture with increasing age (12).

The most important finding of this study was the high prevalence of vitamin D deficiency in elderly population. From the 193 subjects, 129 (76.8%) had serum vitamin D level less than 20ng/ml and 90.05% has vitamin D level below 30ng/ml, while vitamin D measurements in all subjects were done during spring and summer, when sunshine exposure is optimal. In one multinational study of 18 countries in various latitude regions, the mean values for vitamin D were highest in Latin America (29.6 ng/ml) and lowest in the Middle East (20.4ng/ml) despite a favorable latitudes (13).

According to the new IOF audit, hypovitaminosis D was prevalent (>50% of groups studied) throughout the middle east region (1). The mean vitamin D level in patient with osteoporosis in our study was 19.78 ng/ml, which is less than the mean vitamin D status in elderly people with normal BMD (20.46 ng/ml), but this difference is not statistically significant.

Because of secondary hyperparathyroidism due to low vitamin D level, most studies show a positive association between serum vitamin D level and bone density (14-16), but this relation does not show in few studies like our research (17-18). These differences may have some reasons; correlation in studies which shows this relation is seen in distinct levels of serum vitamin D level, and some of these studies show this correlation in special anatomic region, for example, the lumbar vertebra (16). Calcium and vitamin D supplementation also can influence on this relationship. It seems higher doses of calcium and vitamin D supplementation can change this association (19) but we did not characterize the use of supplementation in the subjects. Because of high percent of subjects in vitamin D deficient group in our study (76.8%), examining with other subgroups could not be done.

Although we did not find any relationship between bone mineral density and serum vitamin D level, deleterious effects of vitamin D deficiency on the bone, fracture risk, balance and falling incident are widely accepted (20-23) and the recent data suggest that vitamin D deficiency may play a role in other conditions, including neurological and cardiovascular disorders (24-26). Because of different roles of vitamin D in skeletal and nonskeletal health, this problem needs more attention. In conclusion our findings revealed higher prevalence of osteoporosis in elderly people especially in women compared to other studies in Iran, and also showed high prevalence rate of vitamin D deficiency. No relationships were found between serum vitamin D and bone density in this study. 

## Notes

**Funding: **This project was supported by the deputy of research and Technology in Babol University of Medical Sciences (grant No 892917).
